# Evaluating physicochemical properties of crude oil as indicators of low-salinity–induced wettability alteration in carbonate minerals

**DOI:** 10.1038/s41598-020-60106-2

**Published:** 2020-02-28

**Authors:** Jin Song, Sara Rezaee, Wenhua Guo, Brianna Hernandez, Maura Puerto, Francisco M. Vargas, George J. Hirasaki, Sibani L. Biswal

**Affiliations:** Department of Chemical and Biomolecular Engineering, Rice University, 6100 Main St., MS-362, Houston, TX 77005 USA

**Keywords:** Environmental chemistry, Geochemistry, Crude oil

## Abstract

The injection of low-salinity brine enhances oil recovery by altering the mineral wettability in carbonate reservoirs. However, the reported effectiveness of low-salinity water varies significantly in the literature, and the underlying mechanism of wettability alteration is controversial. In this work, we investigate the relationships between characteristics of crude oils and the oils’ response to low-salinity water in a spontaneous imbibition test, aiming (1) to identify suitable indicators of the effectiveness of low-salinity water and (2) to evaluate possible mechanisms of low-salinity–induced wettability alteration, including rock/oil charge repulsion and microdispersion formation. Seven oils are tested by spontaneous imbibition and fully characterized in terms of their acidity, zeta potential, interfacial tension, microdispersion propensity, water-soluble organics content and saturate-aromatic-resin-asphaltene fractionation. For the first time, the effectiveness of low-salinity water is found to positively correlate with the oil interfacial tension in low-salinity water. Oils with higher interfacial activity are found to respond more positively to low-salinity water. Moreover, cryogenic transmission electron microscopy images suggest that microdispersion is essentially macroemulsion, and its formation is an effective indicator – but not the root cause – of wettability alteration. The repulsive zeta potential for the rock and the oil in low-salinity water is found to be an insufficient condition for wettability alteration in carbonate minerals.

## Introduction

Reservoir wettability greatly affects the oil recovery process. Carbonate reservoirs, which hold over 60% of the reserved oil in the world^1^, are typically preferentially oil-wet or mixed-wet and highly heterogeneous with natural fractures. The heterogeneity and oil-wetness lead to the low oil recovery efficiency (<30% in average^2^) in carbonate reservoirs because the oil in the matrix cannot be efficiently swept by injection brine and the unfavorable wettability impedes capillary imbibition.

Low-salinity water flooding has drawn increasing attention in the past decade as an emerging low-cost enhanced oil recovery (EOR) technique that improves oil recovery by altering the rock wettability. In this technique, the salinity of the injected brine is reduced, and the ionic composition is often modified. The success of low-salinity water in carbonate systems has been widely reported in core-scale laboratory tests^2–9^ and even field-scale trials^10^. However, the failure of low-salinity water has also been frequently reported^9,11–13^. Therefore, identifying the critical conditions for low-salinity water to alter wettability is extremely important because its effectiveness strongly depends on the specific crude oil/brine/rock system. Numerous studies have investigated the role of brine ionic composition in various crude oil systems. Many recent studies emphasize how the reduction of salinity^4–6,14,15^ and presence of Mg^2+^ and SO_4_^2−^ ^2,3,16–23^ affect the rock-brine interaction by means of core-scale experiments, including spontaneous imbibition^2–4,24,25^ and core flooding^5,11,12,17,19,25,26^ tests. Moreover, fundamental studies have included zeta potential measurements^12,15,27–29^, contact angle measurements^25,30,31^, density function theory calculations^20,21^ and molecular dynamic simulation^14^. Brine composition is typically varied, with the corresponding changes in oil recovery or interfacial properties examined.

However, limited work has been done to systematically demonstrate low-salinity water’s effectiveness as a function of crude oil composition and crude oil properties. Typically, only one or two oils are tested and compared in one specific study of low-salinity water. Jackson *et al*.^12^ tested four crude oils and demonstrated a correlation between zeta potential changes and incremental oil recovery in core flooding. Sohrabi *et al*. conducted a series of studies to investigate the critical condition of the crude oils required for low-salinity water to be effective^7,32–36^. They claim that the incremental oil recovery in low-salinity water for a specific crude oil is related to the capability of the oil to form water-in-oil “microdispersion” with low-salinity water. Their hypothesis was that the formation of water-in-oil microdispersion is a result of the detachment of adsorbed indigenous oil surfactants. The increase in the water content in crude oil after contact with low-salinity water leads to higher desorption of indigenous oil surfactants from the mineral surface leading to wettability alteration and higher incremental oil recovery. Two crude oils, one that forms microdispersion and one that does not in low-salinity water, were tested with carbonate cores via core flooding. Additional oil recovery was observed in the test with the oil forming microdispersion, while no additional oil recovery was observed in the other case, confirming the microdispersion hypothesis. However, the number of oil samples was limited and insufficient to demonstrate a definitive correlation between microdispersion and additional oil recovery. Moreover, the tested crude oils were not fully characterized, and possible correlations with other oil properties were not explored in this research. Finally, the reason microdispersion only forms in some specific oils but not in others has not been clearly explained.

The current study aims to systematically investigate the correlation between crude oil properties and the effectiveness of low-salinity water using a variety of crude oils. Six crude oils and one model oil are fully characterized in terms of oil saturate-aromatic-resin-asphaltene (SARA) fractionation, acidity by the total acid number (TAN), surface activity by the interfacial tension (IFT) in both high- and low-salinity water, electrostatic interactions with the rock (oil/brine and rock/brine zeta potentials), microdispersion propensity (water content in oil) and the water-soluble organics content (organics content in brine after oil contact). Then, all seven oils are tested in spontaneous imbibition experiments with Indiana limestone core samples at an elevated temperature to investigate the oils’ response to low-salinity water as a function of their characteristics. With the findings on correlations between an oil’s characteristics and its response to low-salinity water, we identify indicators for the effectiveness of low-salinity water and evaluate possible mechanisms for low-salinity-water–induced wettability alteration, including rock/oil electrostatic repulsion and microdispersion formation. The nature of microdispersion is also investigated using cryogenic transmission electron microscopy (cryo-TEM) for one of the crude oils. To the best of our knowledge, no existing literature has thoroughly investigated the role of oil characteristics in the effectiveness of low-salinity water with abundant well-characterized oil samples. Our work fills this gap and provides important insights for deciphering the mechanisms of low-salinity-water–induced wettability alteration.

## Methods

Indiana limestone core (Kocurek Industries) is selected as representative of the rocks in carbonate reservoirs. The composition of the rock material is characterized by energy-dispersive X-ray spectroscopy (EDAX), as shown in Table 1, along with other rock properties. As the high-salinity model brine, 5 M NaCl is used, and 0.164 M NaCl, having the same ionic strength as 4 times diluted seawater, is the low-salinity model brine. Brines are made from deionized water (18.2 M Ω•cm) and ACS grade salt. Six crude oils from the Gulf of Mexico (Crude A), Southeast Asia (Crude B) and the Middle East (Crude C-F) are selected for investigation. Saturate-aromatic-resin-asphaltene (SARA) analysis is performed using an improved chromatographic method developed by Rezaee *et al*.^37^, and the TAN (mg KOH/g oil) is measured by an improved ASTM method proposed by Fan and Buckley^38^ to characterize the crude oils. The acids are generally considered more important than bases in studying carbonate wettability because the (positively charged) carbonate surface is attracted to the (negatively charged) acidic species. To further test the effects of asphaltene, we prepare a model oil with only asphaltene as the active component. Asphaltene precipitated from Crude D by pentane is added to a base oil consisting of 87% (vol%) n-dodecane (Sigma-Aldrich, >99%) and 13% toluene (Sigma-Aldrich, >99.8%) to yield Asp0.05 MO model oil (asphaltene content = 0.05 wt%). Thus, seven oils in total are tested and characterized (Table 2).

**Table 1:** Properties of the rock material (Indiana limestone).

Properties	Description
Composition	93.0% CaCO_3_, 3.8% SiO_2_, 1.0% MgCO_3_, 2.2% others
Porosity	16%~18%
Permeability	~50 mD (reported by supplier)
Dimension	Length = 1 inch, Diameter = 1 inch.

**Table 2:** Density, SARA analysis, and total acid number (TAN) of all tested oils.

Test oil	Density (g/cm^3^)	Saturate (wt%)	Aromatic (wt%)	Resin (wt%)	Asphaltene (wt%)	TAN (mg KOH/g)
Crude A	0.899	62.6	16.2	17.0	4.2	0.57
Crude B	0.925	66.3	8.7	25.0	0	0.41
Crude C	0.858	68.9	16.8	13.2	1.1	0.33
Crude D	0.836	70.9	19.0	8.0	2.1	0.06
Crude E	0.852	60.9	20.2	18.3	0.6	0.11
Crude F	0.874	59.6	14.4	22.8	3.2	0.24
Asp0.05 MO	0.766	84.4	15.6	0	0.05	0

The approach to characterize the crude oil responses to low-salinity water relies on spontaneous imbibition at an elevated temperature (90 °C). Spontaneous imbibition is chosen over core flooding for the following reasons: (1) In naturally fractured carbonate reservoirs, the efficiency of viscous displacement is low, and so capillary imbibition is expected to be a primary oil recovery mechanism for oil in a low-permeability matrix; (2) oil recovery in a forced displacement experiment is affected by not only the wettability but also other factors, such as the fluids mobility ratio. Therefore, for demonstrating wettability alteration in carbonate reservoirs, the oil recovery from spontaneous imbibition is more representative than that from core flooding.

To prepare for the spontaneous imbibition test, we first vacuum-saturate Indiana limestone cores with initial high-salinity brine (5 M NaCl) and then saturate the cores with the tested oil by ultra-centrifuging the core immersed in the oil at 11,000 rpm for 48 h to obtain irreducible water saturation. After 24 h of centrifugation, the cores are flipped in the centrifuge cell and centrifuged for another 24 h to eliminate a capillary end effect. The only exception is the case of Asp0.05 MO, for which the centrifuging speed is 9,000 rpm and the time is 5 h. The purpose of the lower speed centrifuging condition is to avoid precipitation of asphaltene from the oil prior to the oil aging and imbibition, as the solvent contains a high percentage of dodecane, a precipitant for asphaltene. The volume of the displaced water during the centrifuging is read *in situ* in the graduated centrifuge cell, and the initial oil saturation (S_oi_) is calculated. The S_oi_ for the Asp0.05 MO is 67%, while the figure ranges from 73% to 83% for the other cases. (See the Supplemental Material S1 for details on the oil saturation for each core.) After oil saturation, the cores are aged with the test oil in individual vessels at 120°C for two weeks to allow the surface-active components from the oil to adsorb on the mineral surface and alter the wettability. After the oil aging, the cores are expected to be mixed-wet, meaning that the surface of large pores with oil contact is oil-wet, while the surface of small pores without oil contact remains water-wet. This wetting state is a reasonable representation of the mixed-wet carbonate reservoirs and the starting point for the wettability alteration experiments. Then, for the spontaneous imbibition testing, the core is put in an Amott cell that has a graduated section to determine volumes. The bottom part of the cell is immersed in a water bath of 90°C, and the displaced oil from the core floats up to the thin, graduated top section, where its volume is read. The cell is first filled with the initial brine, 5 M NaCl. After the oil reading is stable for at least five days, the high-salinity 5 M NaCl brine is switched to low-salinity 0.164 M NaCl brine. The high-salinity brine is first drawn out with a Teflon tubing connected to a syringe. After all the brine is extracted, the low-salinity brine is slowly injected into the cell with another Teflon tubing connected to another syringe. The additional oil recovery is recorded every day until the reading is stable for at least five days. The evaporation of recovered oil is negligible because the top part of the cell remains at room temperature and is long enough to act as a condenser. No noticeable oil volume decline is observed during the experiment. Solution gas drive, an experimental artifact that recovers oil due to the release of vaporized light components from the crude oil, is expected to have only a negligible impact on the analysis of additional oil recovery because (1) it can only affect the initial oil recovery in the high-salinity brine but not the additional oil recovery in low-salinity water, and (2) only dead oils are tested.

The brines (5 M NaCl and 0.164 M NaCl) used in all testing (spontaneous imbibition, zeta potential, IFT, microdispersion ratio, and water-soluble organics content) are pre-equilibrated with calcite powder without air contact to (1) avoid any rock dissolution caused by the contact of fresh brine and (2) avoid significant pH changes during experiments. Therefore, calcite dissolution, an oil recovery mechanism that works in the laboratory but is negligibly effective in the field^39,40^, is inhibited. The equilibrated 5 M NaCl and 0.164 M NaCl brines have pH values of 9.1 and 9.8, respectively. The pH values are higher than those of usual reservoir brines because the model brines do not contain added Ca^2+^, which suppresses calcite dissolution and reduces the pH. The brines are not pre-equilibrated with any crude oils. The brine composition is consistent throughout all experiments, so the results from different experiments can be compared.

To verify the wettability alteration of cores after oil aging, we use the Amott-Harvey wettability index (I_A-H_), a generally employed index to quantify rock wettability, for Crude A, for which the most additional oil recovery is observed in low-salinity water. The I_A-H_ measurement is comprised of four sequential steps: (1) spontaneous imbibition of 5 M NaCl brine starting at irreducible water saturation, the volume of spontaneously displaced oil V_osp_ is recorded; (2) forced imbibition of 5 M NaCl brine, the volume of displaced oil V_of_ is recorded; (3) spontaneous drainage of brine in the tested oil, the volume of spontaneously displaced brine V_wsp_ is recorded; (4) forced drainage of brine in the tested oil, the volume of displaced brine V_wf_ is recorded. The I_A-H_ is calculated using the following equation:

1 $${I}_{A-H}=\frac{{V}_{osp}}{{V}_{osp}+{V}_{of}}-\frac{{V}_{wsp}}{{V}_{wsp}+{V}_{wf}}$$

For reference, 0 < I_A-H_ < 1 indicates preferentially water-wet, while −1 < I_A-H_ < 0 indicates preferentially oil-wet. All steps are performed at room temperature, and the forced imbibition/drainage is achieved by centrifuging the core at 10,000 rpm for 48 h in total (after the first 24 h, the core is flipped to eliminate a capillary end effect).

Electrostatic interaction is generally considered to have a crucial impact on the wetting behavior of carbonates. To examine the effect of the rock/brine and oil/brine zeta potentials on additional oil recovery, we measure the zeta potentials via the electrophoresis method using a light scattering analyzer (DelsaMax PRO). The Indiana limestone core is crushed and ground with a mortar and pestle to produce fine rock powder. The 0.164 M NaCl brine is allowed to equilibrate with the limestone powder in advance in a closed container without air. The pH of the brine after equilibration is 9.8, agreeing with the model calculation result from PHREEQC, an open source geochemical modeling software^41^. For the oil/brine zeta potential, 20 μL oil is added to 20 mL of the rock-equilibrated 0.164 M NaCl solution (pH = 9.8), and the sample is sonicated for 30 s with a probe sonicator (Branson) to allow dispersion. After 45 min, the dispersion sample is extracted and measured. The test brine for the zeta potential measurement is identical (including the equilibration method and pH) to those used in the spontaneous imbibition tests with low-salinity water. The high-salinity 5 M NaCl brine cannot be tested due to the limitation of electric conductivity for the electrophoresis method.

To examine the validity of the microdispersion theory, we also measure the water content in the oils before and after contacting the tested brines. The oil and brine (10 mL in total) are put in contact at a 1:1 volume ratio in a borosilicate pipette with a sealed tip. The pipette is torch-sealed at the top with an acetylene/O_2_ flame after the sample is filled and mounted in a rotisserie-type mixer for end-to-end gentle mixing for 24 h at 6 rpm. The sample is then left quiescent for phase separation for 48 h. Finally, the pipette is snipped at the top, and 3 mL of the test oil is taken from the upper part for water content measurement using a Karl Fischer titration apparatus (model 870 KF Titrino plus). Another Crude A sample prepared by a similar procedure (3 mL in total instead of 10 mL in total; top 0.5 mL instead of top 3 mL is extracted) is used for imaging under cryogenic transmission electron microscopy (cryo-TEM). The oil sample is tested one week after being extracted. Large macroemulsions are allowed to separate due to buoyancy. FEI Vitrobot Mark IV is used for cryo-TEM sample preparation. Liquid nitrogen instead of ethane is used as the cooling media because ethane can react with crude oils. Humidifier is turned off during sample preparation to avoid introducing water from the vapor phase. Standard Quanti-foil cryo-grids do not work well for oil samples possibly due to poor wettability for crude oil. Therefore, 300 mesh Lacy carbon TEM grids are chosen for preparing the samples. To obtain good-quality oil films, both blotting force (1~4) and blotting time (2~10 s) are optimized. The best blotting force on oil sample is 4 and the best blotting time is 6~8 s. Frozen samples are then transferred to Gatan 626 Cryo holder and imaged using JEOL 2011 Cryo-TEM (200 kV, LaB6 filament). Mini Dose System (MDS) is used for image processing.

The interfacial activity of the oils is characterized by measuring the IFT in both the high-salinity 5 M NaCl and low-salinity 0.164 M NaCl brines. The test brines are prepared in two ways: The first approach entails pre-equilibrating with calcite powder, using the same procedure as that for the brines for the spontaneous imbibition and zeta potential measurements. The brine pH is 9.1 (5 M NaCl) or 9.8 (0.164 M NaCl). The second approach is pre-equilibrating with both calcite powder and crude oil (1:1 vol/vol). For the second method, the oil is added to the container after the calcite powder has settled so that the oil does not contact the calcite powder directly. The samples are put on an orbital rotation shaker at 80~100 rpm for at least one week to achieve three phase equilibrium. Moreover, to establish that the crude oils are free of contamination, we also test the IFT in fresh 0.164 M NaCl (pH = 7) brine for all the crude oils.

The organics content in the 0.164 M and 5 M NaCl brine after equilibration with the oils is extracted using dichloromethane (DCM) and characterized by gas chromatography with a flame ionization detector (GC-FID) to investigate the water-soluble surface-active compounds from the crude oils. Filtered 0.164 M NaCl brine (pre-equilibrated with calcite, pH = 9.8) and 5 M NaCl brine (pre-equilibrated with calcite, pH = 9.1) are equilibrated with different oil samples in small vials at room temperature. The samples are put on an orbital rotation shaker to allow phase equilibration. After two days, the brine is extracted and filtered again through a 1μm Teflon filter. After measuring the brine pH, we mix 3 mL of the sample with 1 mL dichloromethane (DCM) using a vortex mixer to extract the organics in the brine. After strong vortex mixing for 1 min, one day is allowed for phase separation and mass transport between phases. The DCM solution containing the organics from the oil is then injected into the GC-FID for measurement. A control sample of just 0.164 M NaCl brine (initial pH = 9.8) or 5 M NaCl brine (initial pH = 9.1) is also tested based on the above procedure. The GC-FID runtime for each sample is 1 h, and the column temperature is 150°C. The DCM peak for all samples is identified at the retention time t = 2.1 min, and the injected DCM amount is determined as the integration of the FID signal between t= 0–8 min. After 8 min, the DCM signal is reasonably weak, accounting for less than 0.1 for the control sample. The integration of the FID signal from t = 8 min to t = 60 min is determined as the total amount of organics extracted from the brine sample. The concentration of extracted organics is defined as the total organics amount divided by the injected DCM amount. Further details can be found in the Supplementary Material (S2).

## Results

### Spontaneous imbibition test of different oils in high- and low-salinity brines

**Figure 1:**
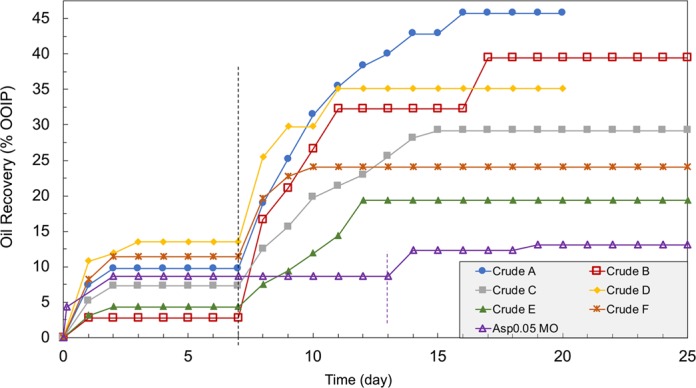
Summary of dynamic oil recovery results for spontaneous imbibition tests at 90°C using different oils. The dashed lines mark the point when high-salinity 5 M NaCl brine is replaced with low-salinity 0.164 M NaCl brine. Both tested brines are pre-equilibrated with calcite.

The oil recovery results for spontaneous imbibition tests of different oils at 90°C are plotted in Fig. 1. In high-salinity 5 M NaCl brine, initial oil recovery is less than 15% for all cases, with figures ranging from 3% to 14%. The relatively low oil recovery in the high-salinity brine is an indication of oil-wetness, confirming the effect of the oil aging process. The Amott-Harvey wettability index I_A-H_ is measured as −0.5 for Crude A, where the highest amount of additional oil recovery is observed. (Details about I_A-H_ measurement are located in the Supplementary Materials [S3].) The negative wettability index further validates that the cores become oil-wet after oil aging. In the wettability index measurement, the residual oil saturation after brine-forced imbibition is less than 5%, which is an indication of mixed wettability because the oil in the larger pores wets the rock surface, meaning that the oil can slowly drain as thin film under centrifugation force, while the smaller pores never have oil invasion. Such wetting behavior represents the typical wettability in carbonate reservoirs. The wettability after oil aging can vary when different oils are tested. Therefore, the core wettability at the start of spontaneous imbibition varies by sample, which is also likely to influence the effectiveness of low-salinity water. However, the role of initial wettability is not the focus of the current work and is not discussed in detail.

**Figure 2:**
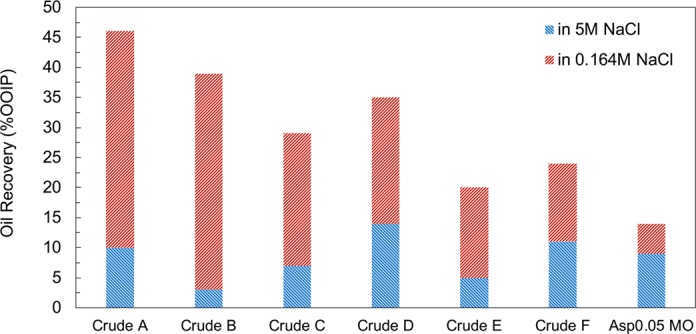
Summary of the oil recovery in high- and low-salinity brines for all cases.

After switching the test brine from the 5 M NaCl solution to the 0.164 M NaCl solution, we observe 5%~36% (of original oil in place) incremental oil recovery. The significantly different oil recoveries demonstrate the role of oil chemistry in determining how effective the low-salinity water is. The origin of such variation is the different oil chemistry because both the rock material and brine compositions are identical across all cases. The final oil recovery results are summarized in Fig. 2. From left to right, oil samples are arranged based on the significance of their response to the low-salinity water. The initial oil recovery in high-salinity water (red columns) does not correlate with the additional oil recovery (blue columns). The additional oil recovery in Fig. 2 is the quantitative characterization of how effective the low-salinity water is in terms of altering the wettability of the oil-wet limestone cores. To examine the possible mechanisms of wettability alteration induced by low-salinity water, we plot oil recovery versus different oil characteristics in the next section to investigate the correlations.

### Correlation between wettability alteration and rock/oil electrostatic repulsion

Electrostatic repulsion between carbonate rock and the attached oil phase in low-salinity water has been widely discussed as a wettability alteration mechanism. Zeta potential measurements of rock/brine and oil/brine interfaces via the electrophoresis method have been the most common approach to characterizing the charge interaction. Therefore, in this work, such measurements are performed for all the tested oils and the Indiana limestone rock in the low-salinity 0.164 M NaCl brine. The Indiana limestone zeta potential in the 0.164 M NaCl brine is measured as −13.6 ± 0.9 mV for a closed system. The negative zeta potential in 0.164 M NaCl is a result of (1) the presence of impurities on the limestone surface and (2) the reduction of cation adsorption compared to high-salinity brines^15^. If the NaCl concentration increases, the Indiana limestone surface will be less negatively charged due to the electrical double layer compression and may become positively charged due to cation (mostly Na^+^) surface binding^15,22^. The Indiana limestone zeta potential is −6.9 ± 2.4 mV in 0.4 M NaCl in a closed system. Measurements for brines of higher salinity are unavailable due to the conductivity limitation of the electrophoresis technique.

**Figure 3:**
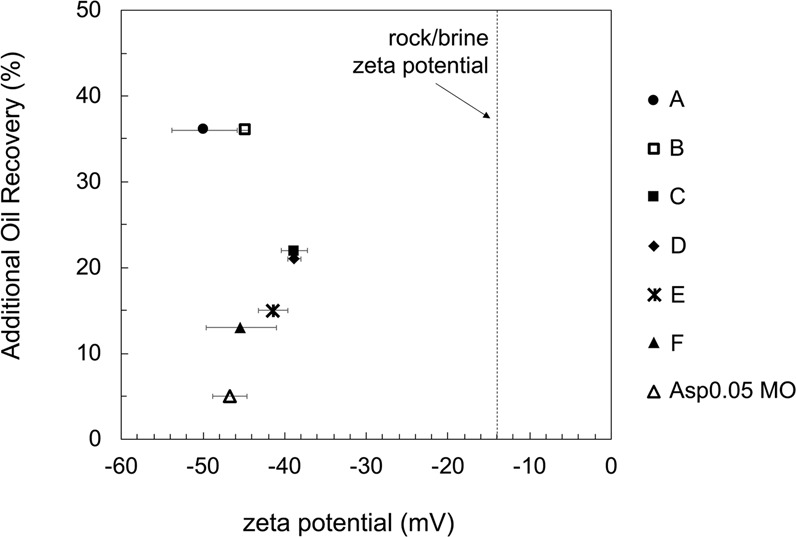
Correlation between the oil/brine zeta potential in 0.164 M NaCl brine and the incremental oil recovery in spontaneous imbibition tests in 0.164 M NaCl brine. The brine in both experiments is 0.164 M NaCl brine pre-equilibrated with calcite (pH = 9.8). The Indiana limestone/0.164 M NaCl zeta potential is −14 mV. Because the rock/brine zeta potential is the same for all cases, the intensity of charge repulsion between oil and rock is expected to be controlled by the oil/brine zeta potential.

Among the different oils, the oil/brine zeta potential should control the electrostatic interaction difference, given the same brine (0.164 M NaCl) and rock (Indiana limestone) surface chemistry. As shown in Fig. 3, all tested oils have strongly negative zeta potentials in the 0.164 M NaCl brine. Therefore, favorable electrostatic repulsion for oil detachment is present in all tested cases. However, no clear correlation is found between the degree of oil recovery and the intensity of oil/rock electrostatic repulsion in terms of the zeta potential. Additionally, the maximum difference between tested oils is only 11 mV, which is insignificant. Even though the electrostatic repulsion favors wettability alteration by contributing to a more repulsive disjoining pressure, other governing factors may play a role in the wettability alteration process.

**Figure 4:**
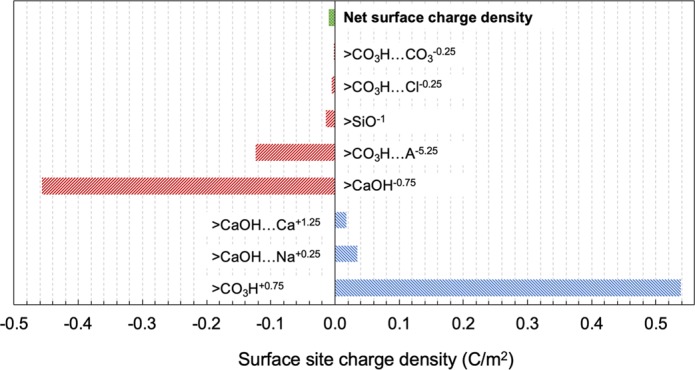
Charge density of Indiana limestone surface sites in 0.164 M NaCl brine in a closed system. Detailed parameters and model descriptions are available in the literature^15^. Specie >CO_3_H…A^−5.25^ represents the surface coverage of organic impurities (A^−6^), and >SiO^−1^ represents the inorganic silica impurity on the natural Indiana limestone.

Moreover, the zeta potential is not a complete description of electrostatic interactions. Unlike sandstone, which has only one type of major surface site (>SiOH), a carbonate surface has two major surface sites, >Ca^+x^ and >CO_3_^−x^ ^15,27,42,43^. The two major surface sites carry opposite charges, and the overall surface charge depends on the ion complexation with them. When the overall rock surface has a negative zeta potential, negatively charged surface species are more abundant than positively charged surface species. However, there are always positively charged surface species (e.g., >CaOH…Ca^+x^)^15,27,43^ present, which can attract the negatively charged carboxylic acids in the oil and prevent the oil from being released from the rock surface. A surface complexation model proposed by Song *et al*.^15^ is used to calculate the surface concentration of charged species on Indiana limestone. Readers are referred to the previous work for the detailed model parameters and the modeling procedure, as the same model parameters and modeling procedure are used here. As described in the previous work, a surface coverage of organic acid impurities (A^6−^) and the presence of silica are assumed to account for the zeta potential difference between natural Indiana limestone and pure calcite. The model predicts the Indiana limestone zeta potential as −7.4 mV in the 0.164 M NaCl brine (closed system), which is 47% lower in magnitude than the measured value of −13.6 mV. Notably, this data point is not included for model parameter fitting. Based on the model calculation, the charge density of surface sites on Indiana limestone in low-salinity 0.164 M NaCl brine (closed system, equilibrated with calcite) is summarized in Fig. 4. That figure demonstrates that abundant positively charged sites may exist on the rock even though the net surface charge is negative. Those positively charged sites can keep the negatively charged oil attached despite the zeta potentials indicating repulsion.

### Correlation between wettability alteration and the microdispersion ratio

“Microdispersion” is a newly proposed term used in recent works to describe the water-in-crude oil dispersions that are found to relate to low-salinity–induced wettability alteration^7,32–36^. The definition is ambiguous because it is not clear whether they are thermodynamically stable microemulsions or kinetically stable macroemulsions. Some researchers relate the formation of microdispersion to the spontaneous emulsification process because ultra-low IFT or mechanical agitation is not required for this process^44–49^. Several mechanisms, including interfacial turbulence and diffusion, have been proposed to explain the spontaneous emulsification in high IFT systems (a few mN/m)^46,49^. To be consistent with the recent literature, we continue to describe such phenomena as microdispersion but examine their nature under cryo-TEM in this section.

**Figure 5:**
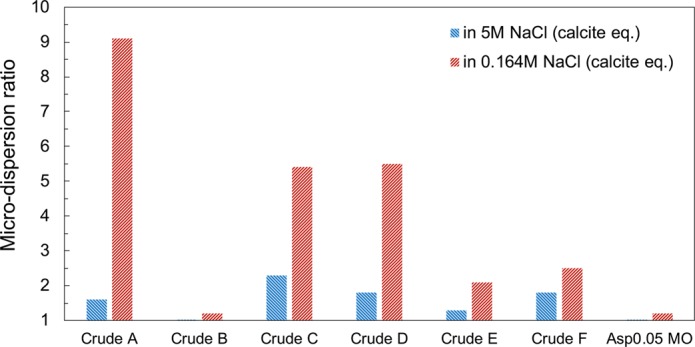
Microdispersion ratio comparison for high- and low-salinity brines. The 5 M NaCl and 0.164 M NaCl brines are pre-equilibrated with calcite for all samples.

The microdispersion ratio is defined as the ratio of the water-in-oil content after oil/brine contact to the original water content in an oil. A ratio equal to unity indicates no microdispersion formation due to water contact. Sohrabi *et al*.^32^ have claimed that the formation of microdispersion alters wettability by releasing the indigenous surface-active components of oil from the mineral surface because those components will move to the newly generated interface of the microdispersion. To evaluate the validity of this mechanism, we measure the microdispersion ratios under both high-salinity (5 M NaCl) and low-salinity (0.164 M NaCl) conditions for all seven oils (Fig. 5**)**. The comparison indicates that microdispersion formation is inhibited under high-salinity conditions. Even for those cases with high ratios in low-salinity brine (Crudes A, C and D), the microdispersion ratios in 5 M NaCl brine are less than 2.5. This observation confirms that microdispersion only significantly appears in low-salinity water. Similar observations for the effect of salinity have been widely reported for water-in-crude oil macroemulsions in the literature^50–53^. The separation efficiency of crude oil and low-salinity water is much lower than that for high-salinity water.

**Figure 6:**
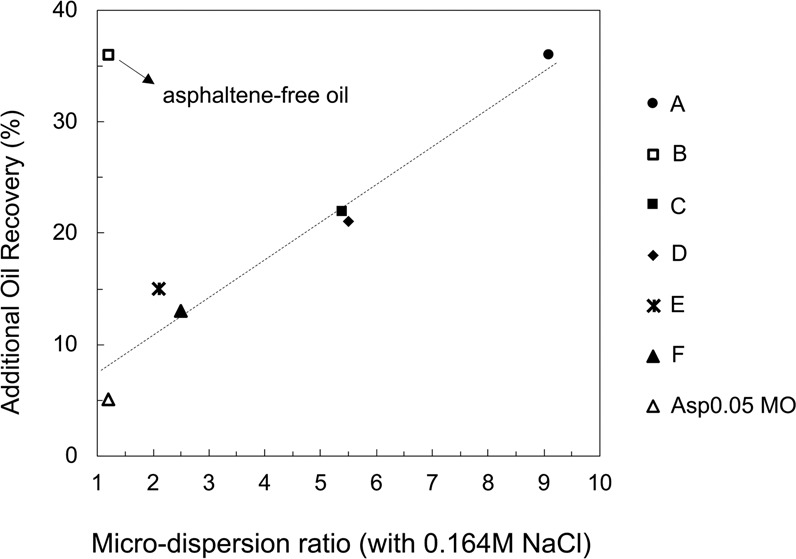
Correlation between the microdispersion ratio of the oils in 0.164 M NaCl brine and the additional oil recovery in spontaneous imbibition tests in 0.164 M NaCl brine. The 0.164 M NaCl brine is pre-equilibrated with calcite before oil/brine mixing. Each point represents the result for one specific oil.

The microdispersion ratio in 0.164 M NaCl brine for all seven oils is plotted versus the additional oil recovery in 0.164 M NaCl brine (Fig. 6). Except for Crude B, an excellent correlation between the microdispersion ratio and additional oil recovery is observed. Linear regression of the six cases (excluding the one outlier, Crude B) achieves R^2^ = 0.95. Those oils with a significantly higher water content after contacting the low-salinity water respond more positively to the low-salinity water in the spontaneous imbibition test. This general trend aligns with the microdispersion hypothesis. However, 36% incremental oil recovery is observed for Crude B, which barely forms microdispersion. Crude B is an oil without any asphaltene. The absence of asphaltene is suspected to be responsible for its low microdispersion propensity.

**Figure 7:**
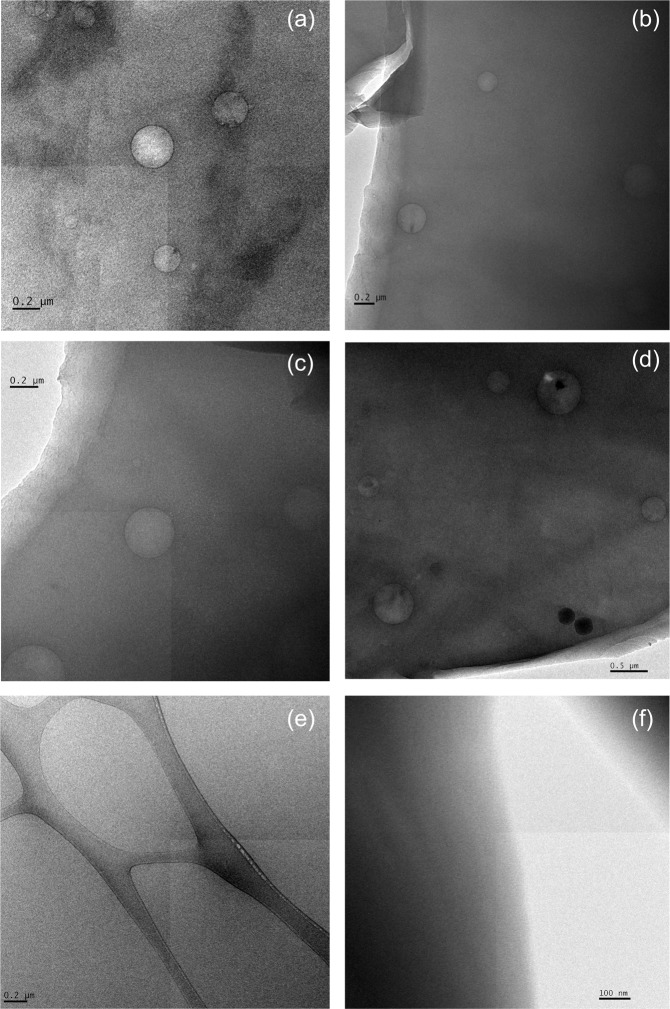
Cryo-TEM images of microdispersions for Crude A oil after equilibration with brines. **(a)~(d)** are images of the oil sample equilibrated with 0.164 M NaCl; **(e,f)** are images of the oil sample equilibrated with 5 M NaCl. No water droplet is found in the oil equilibrated with 5 M NaCl.

To further evaluate the validity of the microdispersion theory, one must understand what microdispersions essentially are and what stabilizes them. Therefore, Crude A oil samples after equilibration with 5 M NaCl or 0.164 M NaCl brine are imaged by cryo-TEM to examine the microdispersion. Figure 7 shows the images of the oil after equilibration with 0.164 M NaCl brine (**[a]~[d])** or 5 M NaCl brine (**[e]** and **[f]**). For the oil after 0.164 M NaCl equilibration, microdispersion droplets ranging from 70 nm (near the center of Fig. 7[c]) to 735 nm (upper right of Fig. 7[d]) in diameter are identified in the cryo-TEM images. As these microdispersion droplets vary significantly in size, they must be macroemulsions instead of microemulsions. Microemulsions have the same droplet size, determined by the thermodynamic equilibrium, and should be smaller than 100 nm. Examples of cryo-TEM images for microemulsions are available in the literature^54–56^. A black ring is present at the interface of water and oil for the droplets. If the black ring were simply a single layer of adsorbed surface-active component, it would not be visible under cryo-TEM at such magnification. Therefore, the ring is suspected to be the adsorbed layer of the asphaltene aggregates. The presence of this black ring for all observed emulsion droplets demonstrates the importance of asphaltene in stabilizing microdispersions. These images support the hypothesis that Crude B’s limited microdispersion is due to the lack of asphaltenes. Microdispersion cannot be found in the oil equilibrated with 5 M NaCl brine even after several trials (Fig. 7[e,f]). The oil equilibrated with 5 M NaCl has so little microdispersion that it is unlikely to be found by TEM.

The outlier case of Crude B indicates that microdispersion formation is not a necessary condition for wettability alteration. Moreover, the formation of microdispersion does not require the release of adsorbed surface-active components on the mineral surface. The original microdispersion theory assumes that the desorption of surface-active components from the existing oil/water interface is required to form a new oil/water interface of microdispersions. However, it ignores that the majority of surface-active components are present in the bulk oil as reverse micelles rather than staying within the limited area of the oil/water interface. Therefore, there are abundant surface-active components in the bulk oil to form microdispersions, and the removal of these components from the mineral surface is unnecessary. Microdispersion formation is unlikely to be the root cause of wettability alteration. However, given the correlation in Fig. 6, microdispersion is a good indicator of the low-salinity water effect if asphaltene is present in the oil, and it likely relates to the intrinsic oil characteristics that govern low-salinity–induced wettability alteration.

**Figure 8:**
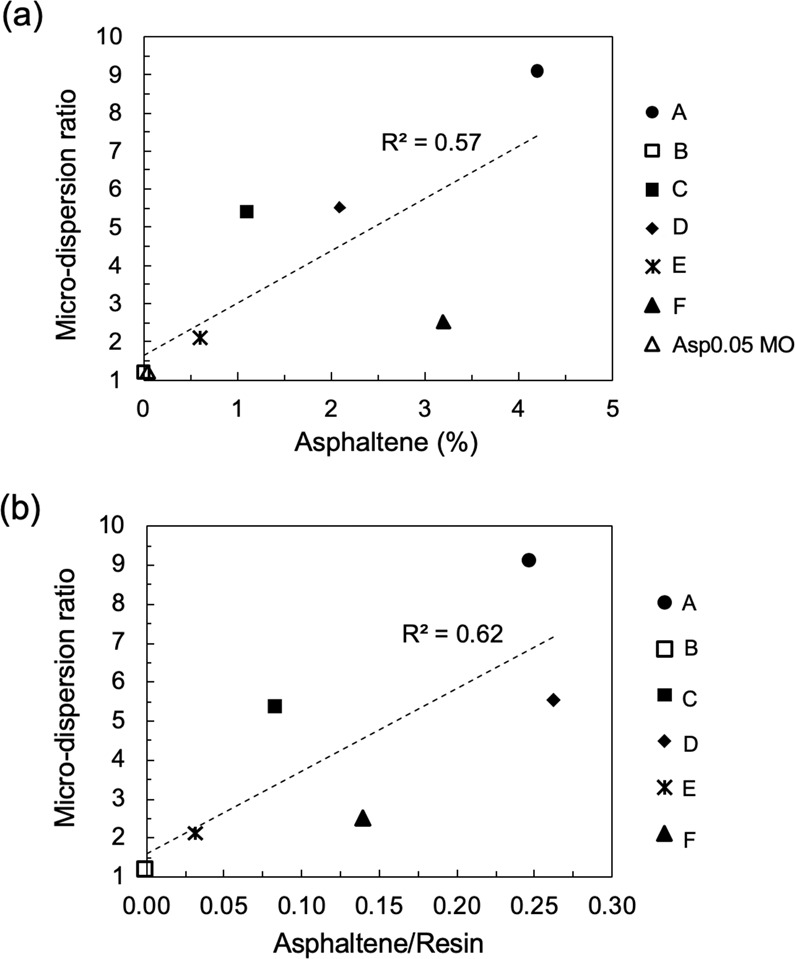
Effect of asphaltene presence on the microdispersion ratio. The microdispersion ratio plotted versus (**a**) the asphaltene content for all oils and (**b**) the asphaltene/resin ratio for all oils with resin fraction. Each point represents one specific oil.

To check if asphaltene is a determining factor for microdispersion formation, we plot the microdispersion ratio versus the asphaltene content and asphaltene/resin ratio in Fig. 8. The asphaltene/resin ratio evaluates the asphaltene instability because resin stabilizes asphaltene in crude oil^57–61^. A high asphaltene/resin ratio typically indicates asphaltene being unstable. The trend in Fig. 8 illustrates a generally positive correlation between the microdispersion ratio and the asphaltene content (a) or asphaltene instability (b), even though the linear regression R-squared value is poor. The asphaltene/resin ratio has a slightly stronger correlation with microdispersion formation (Fig. 8[b]; R^2^ = 0.62). The general trend agrees with the widely accepted premise that precipitated asphaltene can stabilize water-in-oil dispersion, probably due to the formation of an asphaltene rigid layer at the oil/water interface and its ability to provide electrostatic and steric repulsion for a repulsive disjoining pressure^62–65^. Duboue et al^49^. have found de-asphaltenated crude oil to have less microdispersion compared to the original crude oil, even though microdispersion still exists for the de-asphaltenated oil. Apparently, the correlation is not strong enough to conclude that asphaltene instability is the only governing factor for microdispersion formation. In addition to asphaltene, naphthenic acids and their soaps also stabilize emulsions^64^. We attempt to quantify the effect of naphthenic acids by correlating the TAN and the microdispersion ratio. However, the correlation is still unsatisfactory (not shown in figures). The likely reason is that because the TAN also measures acidic compounds that are too hydrophobic to be active at the oil/water interface, it cannot effectively characterize the content of naphthenic acids and their soaps^64,66^.

In sum, the microdispersion formation is confirmed to be an important indication of the low-salinity-water–induced wettability alteration from Fig. 6. However, wettability alteration may still be triggered without microdispersion formation (e.g., Crude B). The absence of asphaltene in Crude B is likely responsible for its low microdispersion propensity (Fig. 8**)**. The intrinsic mechanism of wettability alteration must involve interactions with the solid phase. Thus, microdispersion formation as a result of the fluid-fluid interaction, does not directly influence the wettability of the mineral. However, according to the correlation in Fig. 6 microdispersion formation serves as a good indication of the extent of wettability alteration.

### Correlations between wettability alteration and oil interfacial tension in low-salinity water

**Figure 9:**
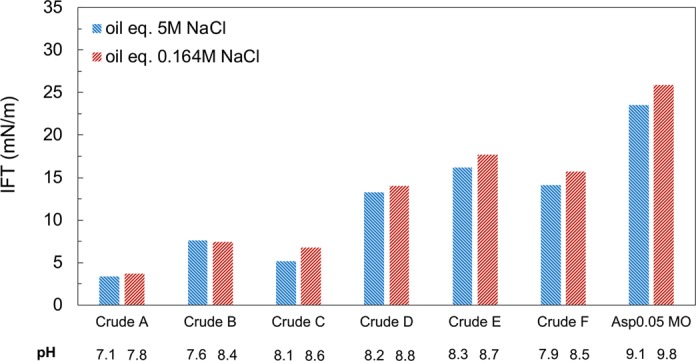
IFT comparison between high-salinity and low-salinity conditions for all tested oils. The brines are pre-equilibrated with calcite and the tested oil (1:1 vol/vol) for all cases. The IFT is also measured using calcite-equilibrated brines without oil equilibration. In that way, the 5 M NaCl brine has a pH of 9.1, and the 0.164 M NaCl brine has a pH of 9.8. Results are generally similar (<2 mN/m difference), with a few exceptions (up to 4.8 mN/m difference), and are reported in the supportive material.

The interfacial activity of oils is hypothesized to relate to the wettability alteration process. The reason for this hypothesis is that Crude B, an oil that has a limited microdispersion propensity but that responds significantly to low-salinity water, is known to be a surface-active oil from our experience. The IFTs in high- and low-salinity brines are compared in Fig. 9 for all seven oils. The tested brines and oils, as well as calcite powder, are pre-equilibrated before the measurements to ensure reproducible and representative IFT values. No significant change in the IFT is observed for any of the seven oils when comparing measurements in high- and low-salinity brines. This indicates that the reduction of capillary pressure, as in surfactant EOR, is not the cause of additional oil recovery in low-salinity water. Interfacial tension is also measured in an alternative way using brines equilibrated with calcite only. In this method, the brine has an identical pH of either 9.1 (5 M NaCl) or 9.8 (0.164 M NaCl) for all oils, and the pH aligns with that in the spontaneous imbibition test. The IFT values measured in the alternative way are provided and compared with the values in Fig. 9 in the Supplementary Material (S4). Only minor differences are observed when comparing the two methods, and the general trend remains the same.

The IFTs of the oils range from 3.7 mN/m to 25.9 mN/m in the 0.164 M NaCl solution pre-equilibrated with rock and oil. The presence of calcite increases the brine alkalinity and promotes soap generation, which results in a lower IFT compared to the IFT in fresh brine (pH = 7). To confirm that the low IFT does not come from surfactant contamination of the oils, we also measure the IFT for all crude oils in fresh 0.164 M NaCl brine (see Supplementary Material S4). The IFT in fresh brine quickly reaches steady state, and the value ranges from 15 mN/m to 27 mN/m, which are reasonable for crude oils.

**Figure 10:**
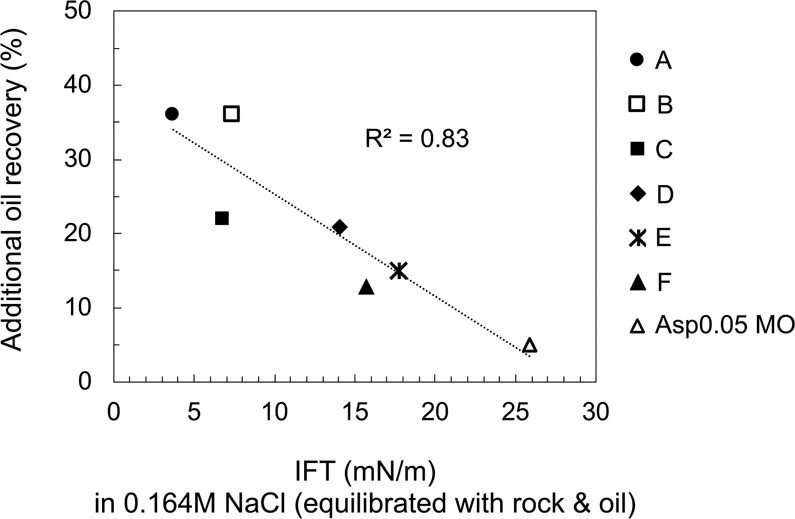
Correlation between the IFT of oils in 0.164 M NaCl brine and the additional oil recovery in a spontaneous imbibition test in 0.164 M NaCl brine. The 0.164 M NaCl brine is pre-equilibrated with calcite and the tested oil (1:1 vol/vol) for all cases. Each point represents the result of one specific oil.

A strong correlation is found between the oil IFT in low-salinity brine and wettability alteration in Fig. 10, with an R^2^ = 0.83 for the linear regression. Crude oil B, which does not form microdispersion but has a low IFT and responds positively in low-salinity brine, is not an outlier in Fig. 10. We also attempt to correlate wettability alteration and the amount of asphaltene or total acidic components, both of which can potentially contribute to oil surface activity. However, no conclusive trend is found in terms of correlating wettability alteration with either the asphaltene content or TAN, even though oils with higher TANs seem to be generally more responsive to low-salinity water (see Supplementary Material S5). The TAN does not necessarily indicate the number of surface-active components from oil because a portion of the acidic compounds may be too hydrophobic to be active at the oil/water interface^64,66^.

The substantial differences in oil recovery by spontaneous imbibition in Fig. 10 cannot be attributed to a reduction in capillary pressure from a lower IFT oil. The effect of IFT on remaining oil saturation becomes significant only when IFT is reduced by several orders of magnitude (e.g. surfactant EOR)^67^. Therefore, oil recovery by spontaneous imbibition primarily depends on the extent of wettability alteration and not on the small difference of IFT values among different oils. Moreover, wettability alteration is a process involving interactions with the solid mineral while IFT is independent of the solid phase. Therefore, even though correlations are found between wettability alteration and fluid-fluid interactions such as microdispersion formation and IFT, they are manifestations but not the root cause of wettability alteration. Young-Laplace equation (Eq. 2) can be applied to assist the analysis on wettability:

2 $$\cos \,\theta =\frac{{\gamma }_{so}-{\gamma }_{sw}}{{\gamma }_{ow}}$$

$\theta$ is the contact angle measuring through the water phase. ${\gamma }_{ow}$ denotes the oil/water IFT (mN/m). ${\gamma }_{so}$ and ${\gamma }_{sw}$ are the surface energy (mN/m) of the mineral in oil and water, respectively. ${\gamma }_{ow}$ has been shown to be almost unchanged after brine salinity reduction. If the limestone surface becomes more water-wet (smaller $\theta$), either it has a lower surface energy in the water (${\gamma }_{sw}$), or it has a higher surface energy in the oil (${\gamma }_{so}$), or both. Therefore, solid-fluid interaction must be involved. Further investigations are required to understand the underlying mechanism of low-salinity-induced wettability alteration.

### Oil interfacial activity as an indicator: interfacial tension, microdispersion and water-soluble organics

After oil/brine contact, the majority of surface-active components from crude oils stay in the bulk oil and assist in forming microdispersion, and part of these components adsorb at the oil/brine interface and lower the IFT. Moreover, a low percentage of the surface-active components are relatively hydrophilic and can partition into the brine. In the previous discussion, both the IFT and microdispersion ratio – characterizing surface-active components at the interface and in the bulk oil, respectively – are shown to correlate with low-salinity–induced wettability alteration. Therefore, we can assume that the water-soluble organics content of crude oils may also show a similar correlation.

**Figure 11:**
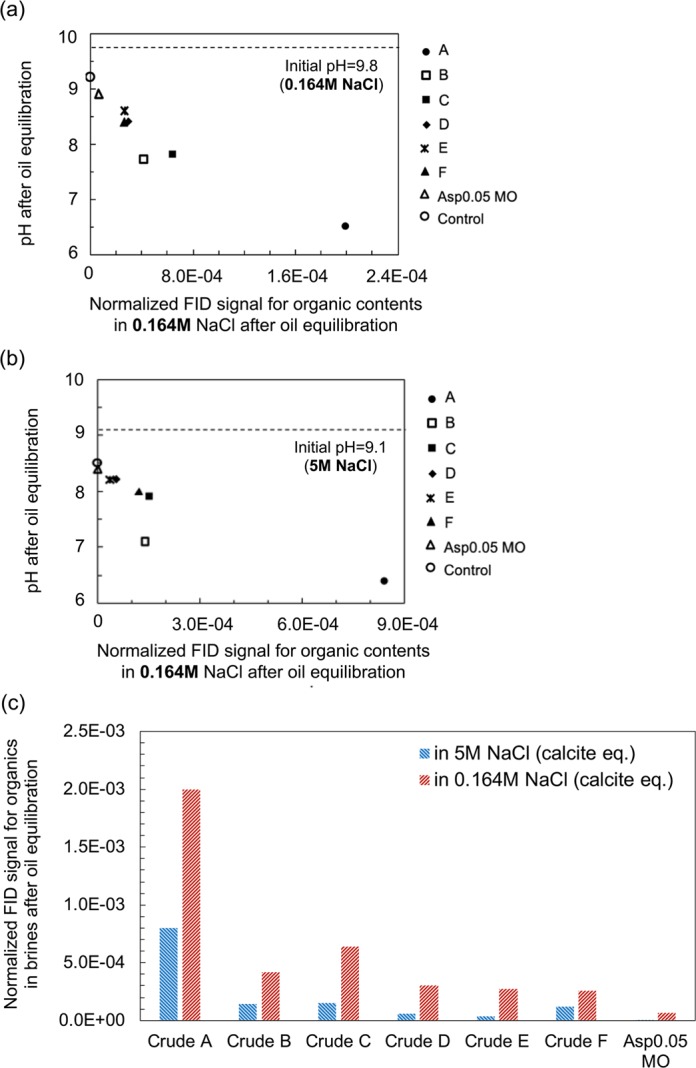
Normalized flame ionization detector (FID) signal for the water-soluble organics content of oils in high- and low-salinity brines. (**a**) Organics content in 0.164 M NaCl brine after oil equilibration plotted versus the brine pH after brine/oil equilibration. The initial pH of the 0.164 M NaCl brine (pre-equilibrated with calcite powder) is 9.8. (**b**) Organics content in 5 M NaCl brine after oil equilibration plotted versus the brine pH after brine/oil equilibration. The initial pH of the 5 M NaCl brine (pre-equilibrated with calcite powder) is 9.1. (**c**) Comparison of the water-soluble organics content in 5 M NaCl brine and 0.164 M NaCl brine for all samples.

In this section, another new indicator, the water-soluble organics content in the oil, is first identified and then compared with two other indicators: the microdispersion ratio and IFT. We use GC-FID to characterize the water-soluble organics content of the crude oils. The absolute value of the organics concentration in the brine is hard to measure because hundreds of different surface-active organic components of the crude oil may partition into the aqueous phase, and no standard calibration curve can be made for them. Therefore, the concentration of the organics in the sample is defined as the integration of the FID signal for all the organics except solvent, divided by the solvent amount. The FID signal as a function of the retention time for all the samples is available in the Supplementary Material (S2). This so-called normalized FID signal for organics content is plotted versus the pH of the brine after oil contact to validate the FID measurement (Fig. 11[a,b]). Since the initial brine is basic (pH >9), primarily the acidic compounds in oils are extracted into the aqueous phase. Therefore, a more significant pH drop should be observed for those oils with higher water-soluble organics levels in the tested brine.

Figure 11[a,b] demonstrates a negative correlation between the drop in the brine pH after oil contact and the organics content in the brine measured by GC-FID. This trend validates the GC-FID measurements of the organics content in the brine because the decline in the brine pH indicates the partition of surface-active carboxylic acids into the aqueous phase from the oil. The pH of the control samples (9.2 for 0.164 M NaCl brine, 8.5 for 5 M NaCl brine) is slightly lower than their initial pH (9.8 for 0.164 M NaCl brine, 9.1 for 5 M NaCl brine) after being left in the vial for two days without oil contact. The drop from the initial pH is attributed to the contact with the small amount of air (400 ppm CO_2_) trapped in the sample vial.

Figure 11[c] compares the water-soluble organics content in high- and low-salinity brines for all tested samples. For each oil, the peaks for water-soluble organics are similar in shape but different in intensity if one compares the results in high- and low-salinity water (see Supplementary Material S2). The organics content in 5 M NaCl brine after oil equilibration is lower than that of the 0.164 M NaCl brine for all the cases, similar to the trend seen when comparing the microdispersion ratio in high- and low-salinity brines in Fig. 6. Similar phenomena have also been observed in the literature when Na_2_CO_3_ is used to tune salinity and the active soap number of oil is measured by titration methods^66^. Water-soluble acids in crude oils stay in the oleic phase at high ionic strength but partition into the aqueous phase at low ionic strength. Several factors may be responsible: (1) Charge repulsion is much stronger in low-salinity conditions between the headgroups of surface-active components in oils. This can reduce the curvature of water-in-oil microdispersion and even change the sign of the curvature. Therefore, swollen micelles in the aqueous phase may form in low-salinity conditions. (2) Low-salinity brine after calcite equilibration has a higher pH (9.8) than high-salinity brine (pH = 9.1), which means that acidic components are more deprotonated and have higher solubility in low-salinity water. (3) The higher polarity of the high-salinity brine may inhibit the partitioning of organics from the oil phase and result in the lower organics content in high-salinity brine.

**Figure 12:**
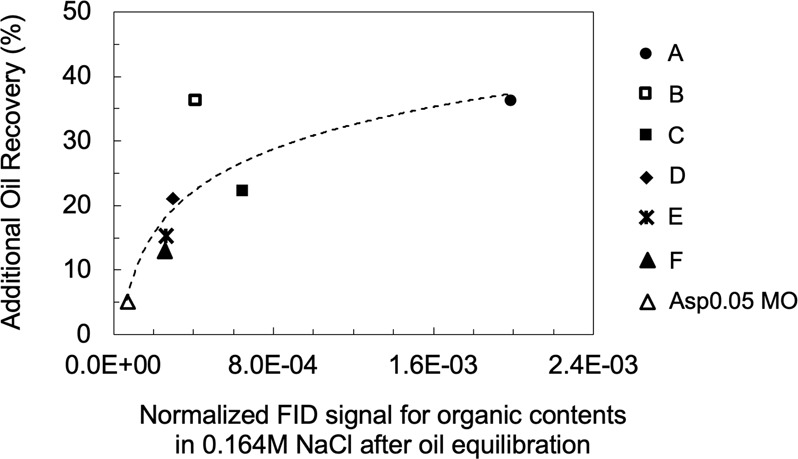
Correlation between the water-soluble organics content in 0.164 M NaCl brine after oil equilibration and additional oil recovery in 0.164 M NaCl brine for a spontaneous imbibition test. Each point represents the result of one specific oil.

A generally strong correlation is observed in Fig. 12, indicating that the extent of wettability alteration is aligned with the quantity of water-soluble components from the oil. The water-soluble compounds in the oil indicate the oil/water interfacial activity because they must contain a hydrophilic portion giving rise to their solubility in the aqueous phase, so they are surface-active at the oil/water interface. Similarly, the microdispersion formation in the oil phase can also suggest oil surface activity because surface-active components are required to stabilize the dispersion of water in oils. In this sense, it is reasonable to find a similar trend in Figs. 6 and 12.

**Figure 13:**
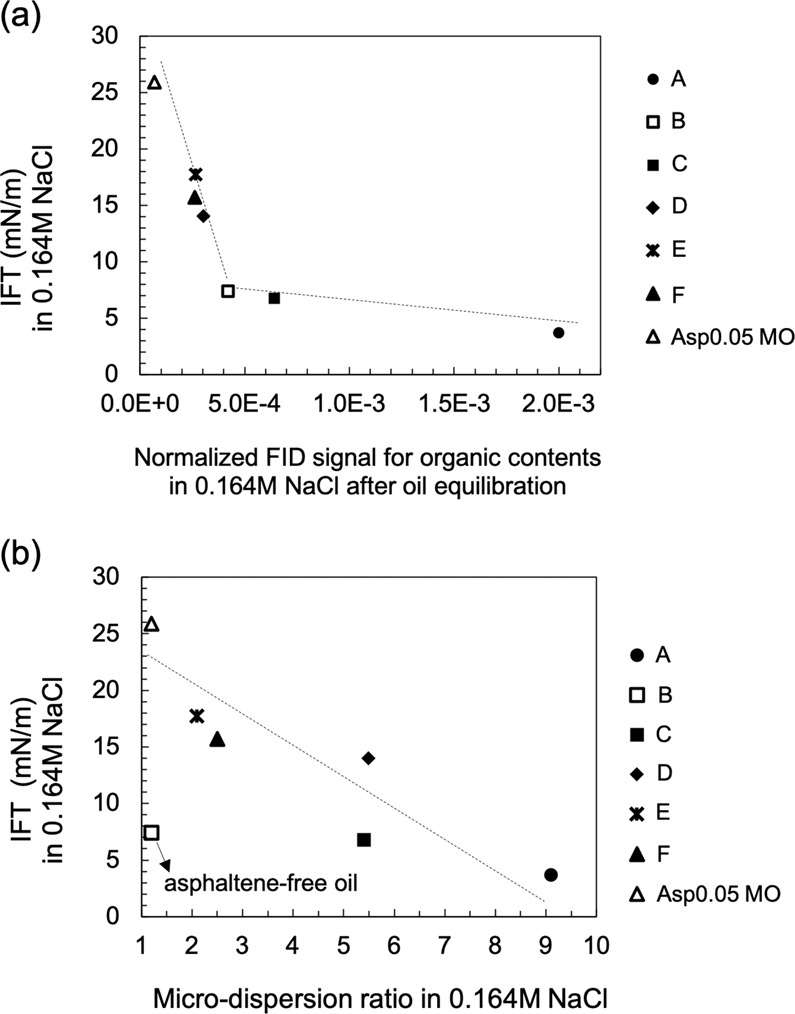
Relationship between IFT and the other two indicators: microdispersion and water-soluble organics. (**a**) The IFT in 0.164 M NaCl brine plotted versus the normalized FID signal for the organics content in 0.164 M NaCl brine after oil equilibration for all oils. (**b**) The IFT in 0.164 M NaCl brine plotted versus the microdispersion ratio in 0.164 M NaCl brine for all oils. The IFT is measured using the brine pre-equilibrated with rock and oil (1:1 vol/vol). Each point represents one specific oil.

Interfacial activity (IFT), microdispersion formation and the water-soluble organics material in the crude oil are three indicators found to correlate with wettability alteration in low-salinity brine. The normalized FID signal shown in Fig. 12 characterizes the more hydrophilic surface-active components partitioning into the aqueous phase, and the microdispersion ratio in Fig. 6 characterizes the more hydrophobic surface-active components forming swollen reverse micelles in the oleic phase. To shed light on the relationship between the indicators, we plot the IFT in low-salinity brine versus the other two indicators in Fig. 13. The general trend indicates that more surface-active oils have more water-soluble organics material (Fig. 13[a]). If we consider the various crude oils analogous to a base oil containing different concentrations of surface-active components, then the change of slope in Fig. 13[a] appears to indicate a critical micelle concentration (CMC) for the water-soluble organics material. This value may be called a “pseudo-CMC” because there are hundreds of different natural surface-active components in each oil, and the composition of crude oils can vary significantly. The pseudo-CMC illustrates how water-soluble organics form micelles in low-salinity brine for Crudes A, B and C, but not Crudes D, E and F. The pseudo-CMC may not be relevant to the existence of microdispersions, which are stabilized by other relatively more hydrophobic surface-active components residing in the oleic phase. However, more surface-active oils do have more water-in-oil microdispersions, as shown by Fig. 13[b]. One significant outlier is Crude B, which is fairly surface-active but does not contain asphaltenes. The plots in Fig. 13 demonstrate that both water-soluble organics and the microdispersion ratio can be treated as indirect measurements of an oil’s interfacial activity in brines (with potential outliers such as Crude B). Therefore, we hypothesize that the intrinsic mechanism of low-salinity–induced wettability alteration is related to the crude oil interfacial activity (or interfacial energy of the oil/brine interface). Although the microdispersion ratio in low-salinity water is generally a suitable indicator, it is probably not intrinsically relevant to the wettability alteration mechanism. Instead, it is an indicator only because it correlates with the interfacial activity (Fig. 13[b]).

## Conclusions

Six crude oils and one model oil have been fully characterized and utilized in spontaneous imbibition tests to investigate the relationship between the oil properties and the response of the oil to low-salinity water in a carbonate system. Possible indicators and mechanisms for wettability alteration in low-salinity water are examined by investigating the correlations between the oil recovery results via spontaneous imbibition and oil characteristics, including the oil/brine zeta potential, microdispersion propensity, asphaltene content, asphaltene instability, IFT and water-soluble organics content.

The oil IFT in the low-salinity water is found to greatly influence the low-salinity–induced wettability alteration process. This finding is directly supported by the strong correlation between the oil/brine IFT in low-salinity water and incremental oil recovery via spontaneous imbibition (Fig. 10). It is also indirectly supported by the investigations of microdispersion propensity and the partition of water-soluble organics into the aqueous phase, both of which are promoted by surface-active components in the oil (shown in Fig. 13). Microdispersions and the water-soluble organics content serve to characterize the more hydrophilic and the more hydrophobic portion of the surface-active components in the oil, respectively.

Microdispersion formation, which likely requires asphaltene in the oil, is found to be an important indication of wettability alteration in low-salinity water. However, wettability alteration is also observed for an oil that has no asphaltene and fails to form microdispersion. Cryo-TEM images of Crude A after contacting low-salinity water show that microdispersions are essentially macroemulsions. We postulate that microdispersion formation is not a trigger of wettability alteration for the following reasons: (1) The formation of microdispersion is a result of the generation of macroemulsions, which do not require the desorption of surface-active components from rock. There are abundant surface-active components in the bulk oil existing as reverse micelles, which can assist macroemulsion formation; and (2) Crude B, which does not exhibit microdispersion, responds positively to low-salinity water in carbonate minerals.

No correlation is found between the wettability alteration and the oil/brine zeta potential. This observation does not necessarily mean electrostatic interactions are trivial. The repulsive zeta potential for carbonate rock and oil appears to be a necessary, but insufficient condition, for wettability alteration in low-salinity water. Even when the rock/brine zeta potential is negative, positively charged surface species still exist on the carbonate surface, binding to the negatively charged acids from the crude oil.

## Supplementary information

Supplementary Information.
