# Supplementary material for: Evaluating physicochemical properties of crude oil as indicators of low-salinity–induced wettability alteration in carbonate minerals
Source: Sci Rep. 2020 Feb 28;10:3762. doi: 10.1038/s41598-020-60106-2 (PMC7048854; doi:10.1038/s41598-020-60106-2)
Supplement: Supplementary file 1 — Supplementary Information. [file 41598_2020_60106_MOESM1_ESM.pdf]

Supplementary material

Evaluating physicochemical properties of crude oil as indicators of low-salinity–induced wettability alteration in carbonate minerals

Jin Song[†‡], Sara Rezaee[†‡], Wenhua Guo[†], Brianna Hernandez[†], Maura Puerto[†], Francisco Vargas[†], George J. Hirasaki[†]*, and Sibani L. Biswal[†]*

[†]Rice University, 6100 Main St., MS-362, Department of Chemical and Biomolecular Engineering, Houston, TX, 77005 USA.

[‡] These authors contributed equally to the study.

* To whom correspondence should be addressed:
email: biswal@rice.edu, gjh@rice.edu

Email addresses: js110@rice.edu (Jin Song), sr44@rice.edu (Sara Rezaee), whguo@rice.edu (Wenhua Guo), bmh6@rice.edu (Brianna Hernandez), Maura@rice.edu (Maura Puerto), fvargas@rice.edu (Francisco Vargas) , gjh@rice.edu (George J. Hirasaki), biswal@rice.edu (Sibani L. Biswal)

S1. Summary of oil saturations for all the cores in the spontaneous imbibition test

	Sample	ϕ (%)	S_{oi} (%)	S_o (%) 5M NaCl	S_o (%) 0.164M NaCl	Oil recovery in 5M NaCl	Total oil recovery
Centrifuge 11000rpm, 48hrs	Crude A	17.7	77	70	42	10	46
	Crude B	17.1	82	80	50	3	39
	Crude C	18.0	83	77	59	7	29
	Crude D	18.0	80	69	52	14	35
	Crude E	17.1	73	70	56	5	20
	Crude F	17.2	73	65	56	11	24
Centrifuge 9000rpm, 5hrs	Asp0.05 MO	16.1	67	61	58	9	14

S2. Summary of GC-FID measurements for all the tested samples

1. <u>The method to determine organics content from FID signal</u>

To test the water-soluble organics content of an oil, the oil is equilibrated with 0.164M NaCl (pH=9.8) to achieve the equilibrated brine. Then, the water-soluble organic compounds are extracted from the equilibrated brine to DCM for GC-FID testing. The figure below (**Figure S2.1**) shows the summary of FID signal for all tested samples using 0.164M NaCl. The purpose of this figure is to show the consistency of the solvent peak for all measurements (t=2.1mins). The signals for the organics extracted from the brine to DCM are too small to see at this scale.

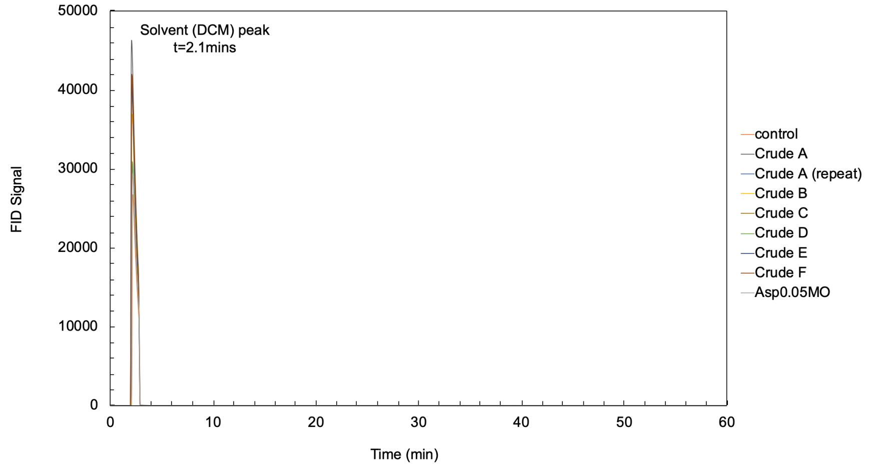

Figure S2.1 Summary of FID signal for all tested cases using 0.164M NaCl.

The figure below (**Figure S2.2**) is to show the FID signal for the control sample case where no signal other than that for the solvent (DCM) is expected for any organics. Based on this plot, the dividing retention time of t=8mins is chosen. Integration of signal before t=8mins represents the amount of solvent. Integration of signal after t=8mins represents the amount of organics extracted from the tested brine (which is equilibrated with the tested oil).

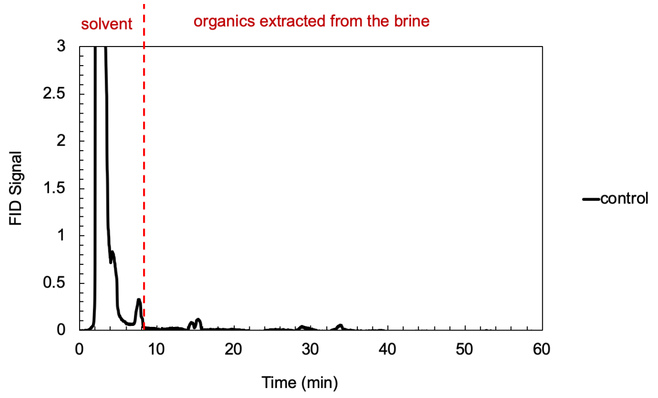

Figure S2.2 FID signal for the control sample using 0.164M NaCl.

For the case of Asp0.05MO, no organics is expected to partition into the brine because asphaltene is the only surface-active component and it is too heavy to be solubilized in the aqueous phase. Therefore, an identical signal as the control sample should be expected for this case of Asp0.05MO. **Figure S2.3** shows consistent result as is expected. It also confirms the stability of the GC-FID signal.

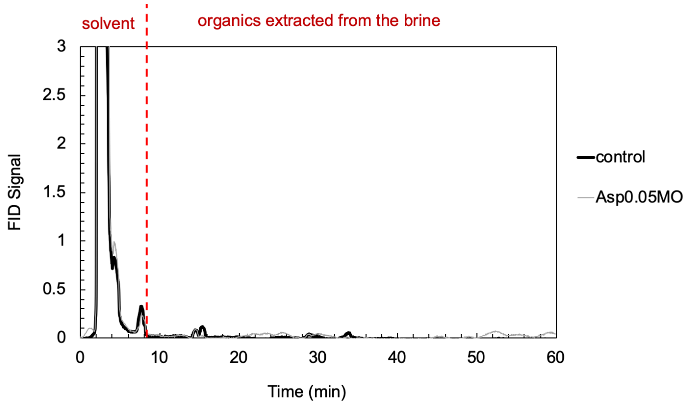

Figure S2.3 FID signal for the control sample and Asp0.05MO sample using 0.164M NaCl.

2. Summary of detailed FID signal for all tested cases in 0.164M NaCl

Figure S2.4 shows the summary of FID signal for all the samples testing with 0.164M NaCl. The sample of Crude A is tested twice to confirm the accuracy of measurement and two measurements agree with each other well after normalizing with the solvent amount.

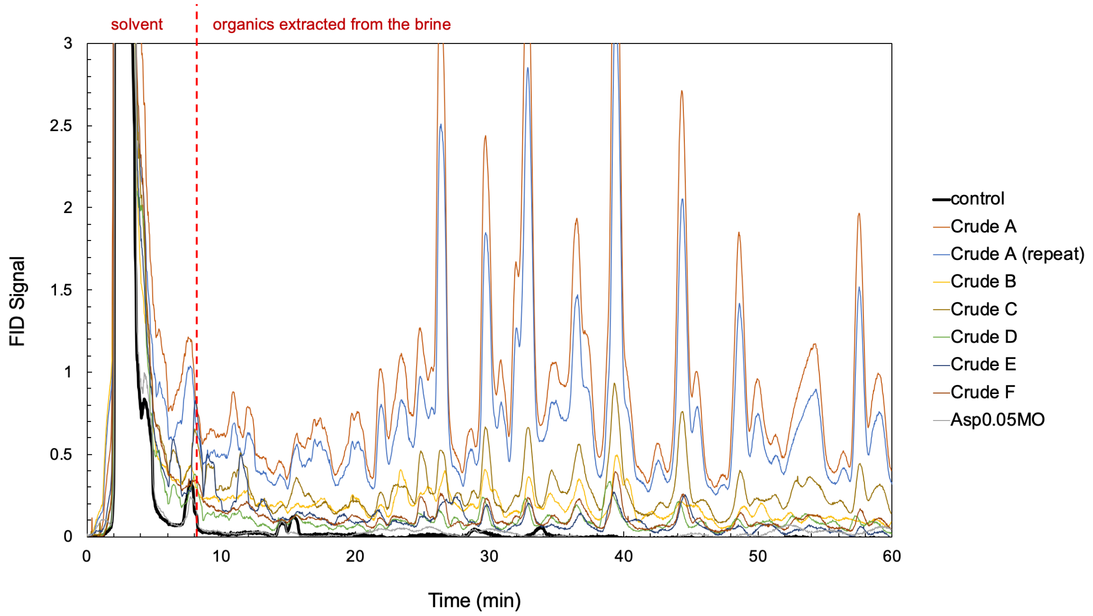

Figure S2.4 FID signal for all tested samples using 0.164M NaCl.

3. FID signal comparison for all tested cases in 5M/0.164M NaCl

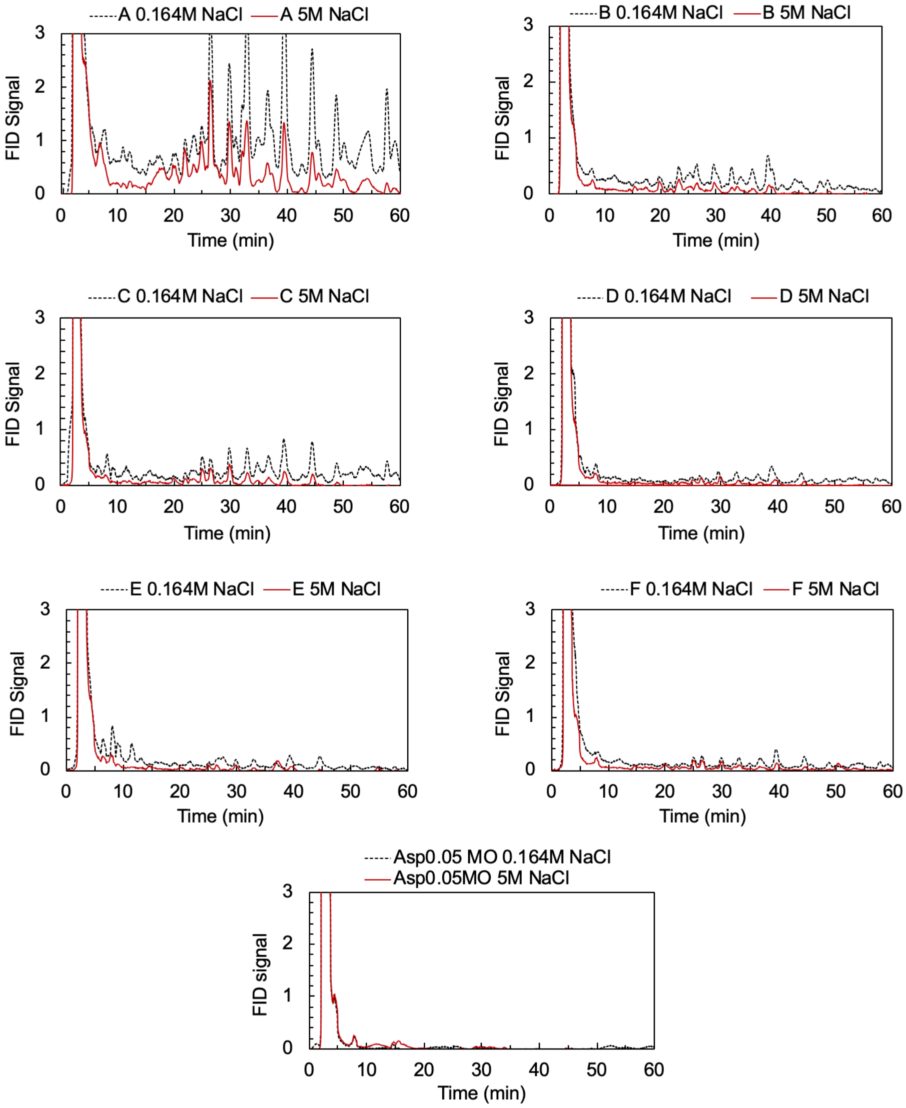

Figure S2.5 Comparison of FID signal for water-soluble organics in 5M NaCl and 0.164M NaCl for all seven samples

S3. Oil saturation in the Amott-Harvey wettability index measurement

The figure below shows the oil saturation of the Indiana limestone core in the process of wettability index measurement. The initial oil saturation is 74%. Very little oil is recovered during the spontaneous imbibition in 5M NaCl (at room temperature). After forced imbibition by ultra-centrifugation at 10000rpm for 48 hours, most of the oil is recovered and the residual oil saturation drops to 5%. Oil saturation increases to 43% after the spontaneous drainage.

Eventually the oil saturation goes back to 71%, roughly the same as initial oil saturation after the forced drainage step.

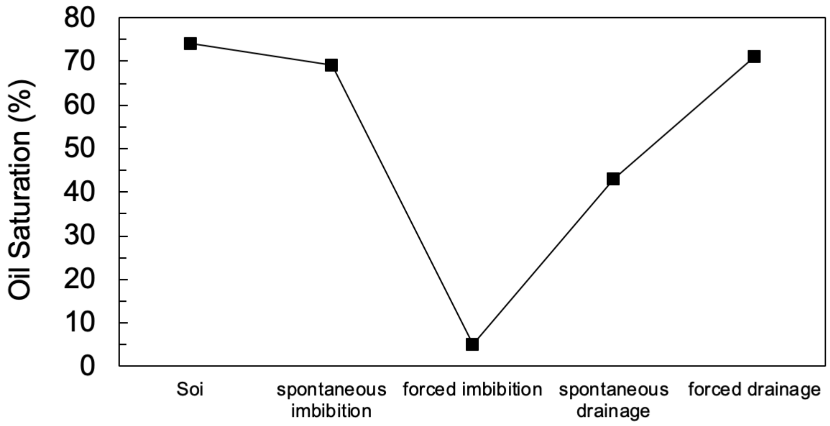

Figure S3.1 Oil saturation of Indiana limestone core during the $I_{A\text{-}H}$ measurement

S4. Effect of equilibration methods on crude oil's IFT

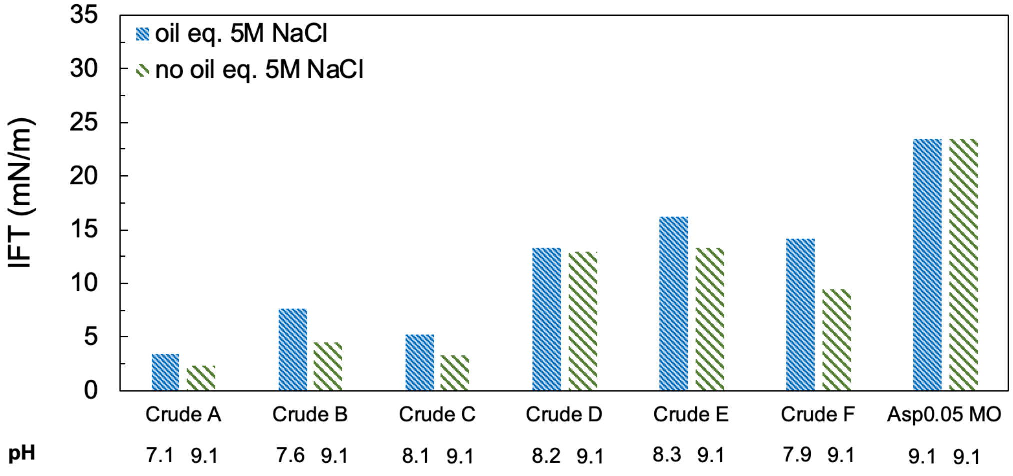

Figure S4.1 Effect of equilibration methods (calcite/brine equilibration or calcite/oil/brine equilibration) on crude oils' IFT in 5M NaCl

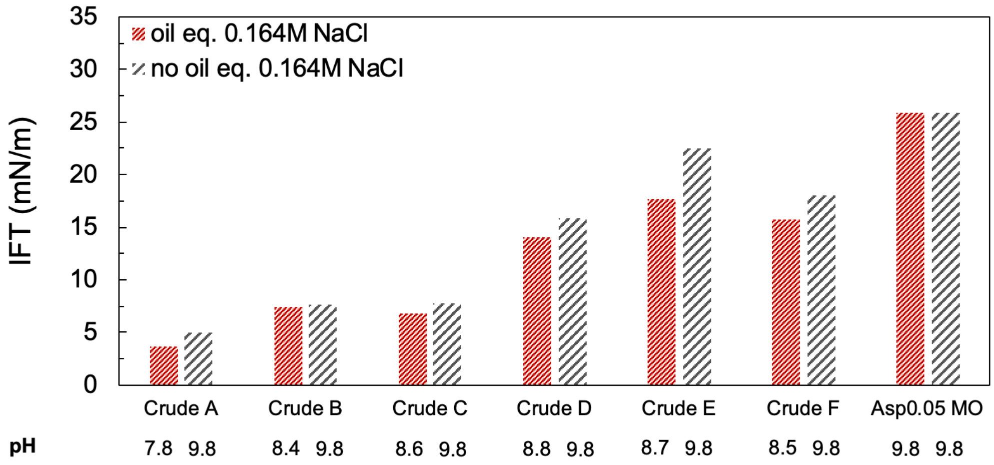

Figure S4.2 Effect of equilibration methods (calcite/brine equilibration or calcite/oil/brine equilibration) on crude oils' IFT in 0.164M NaCl

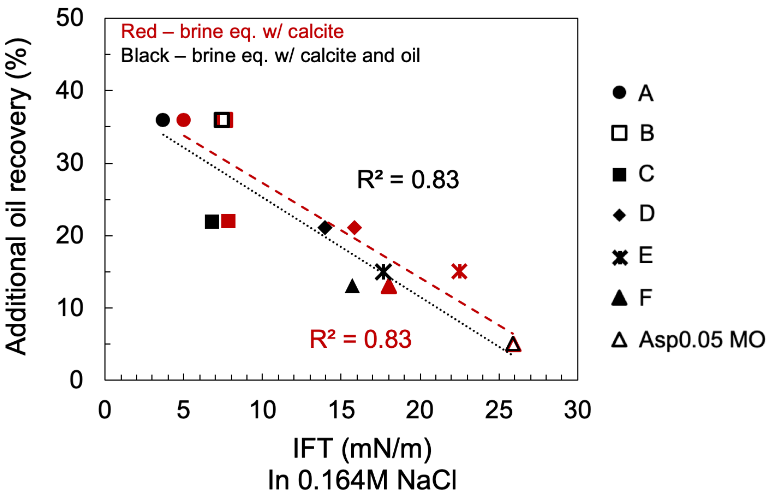

Figure S4.3 Correlation between additional oil recovery and IFT in 0.164M NaCl using IFTs measured in two different ways.

The IFT for all crude oils is measured in 0.164M NaCl brine without any rock equilibration. The values range from 15~27mN/m, which are normal for crude oils, showing that these oils are free of contamination. The IFT for the same oil is lower in the brine with calcite equilibration (pH=9.8) due to soap generation at higher pH.

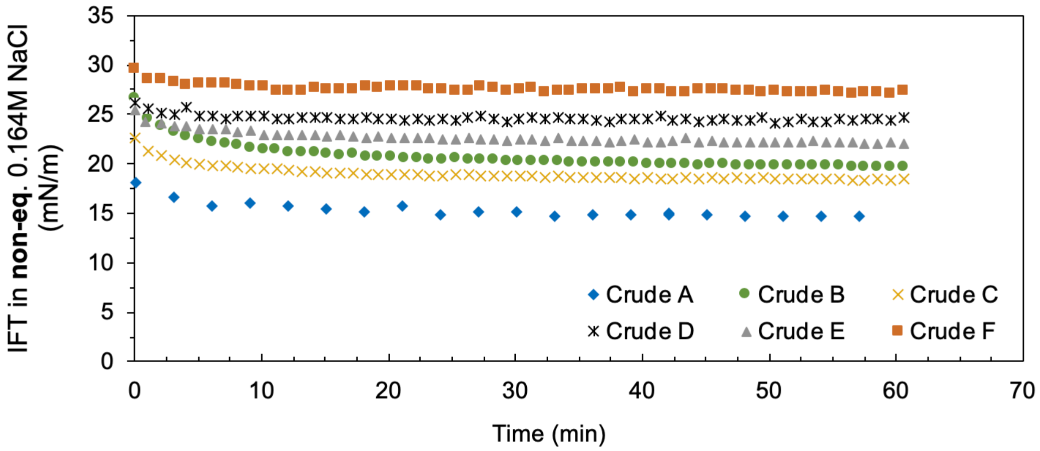

Figure S4.4 Dynamic IFT results of crude oils in 0.164M NaCl without calcite equilibration

S5. Correlations between additional oil recovery in 0.164M NaCl and TAN, asphaltene and asphaltene/resin ratio

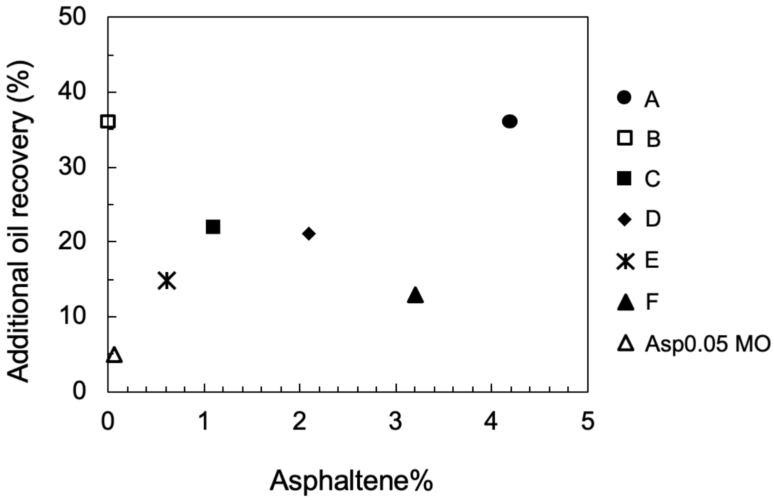

Figure S5.1 Correlation between additional oil recovery in 0.164M NaCl and asphaltene%

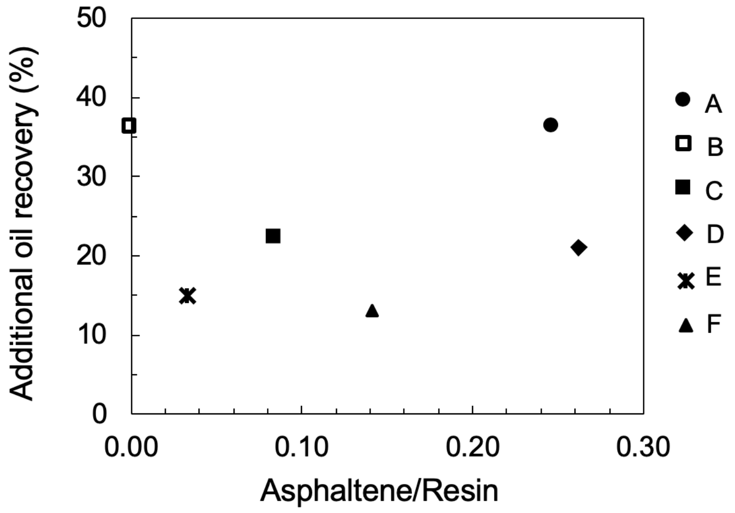

Figure S5.2 Correlation between additional oil recovery in 0.164M NaCl and asphaltene instability

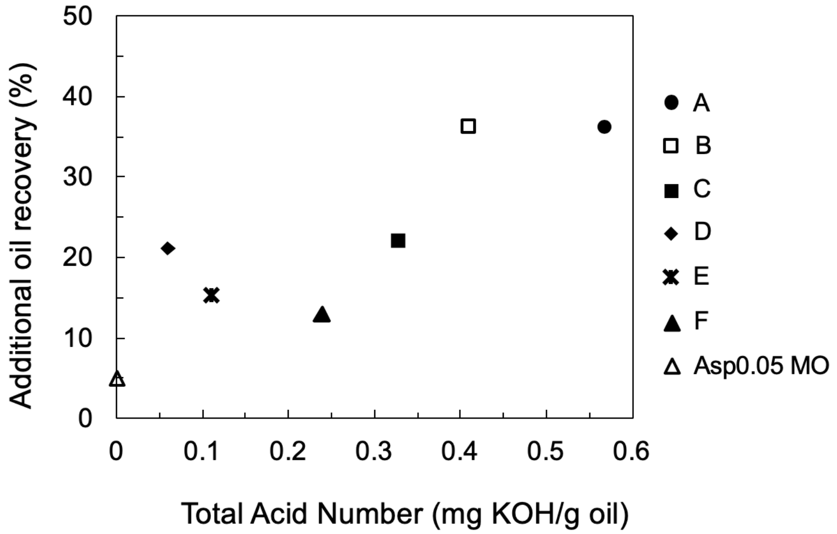

Figure S5.3 Correlation between additional oil recovery in 0.164M NaCl and total acid number (TAN)
